# The Diagnostic and Therapeutic Role of Leptin and Its Receptor ObR in Glioblastoma Multiforme

**DOI:** 10.3390/cancers12123691

**Published:** 2020-12-09

**Authors:** Thomas M. Kinfe, Andreas Stadlbauer, Yavor Bozhkov, Natalia Kremenevski, Sebastian Brandner, Michael Buchfelder, Shafqat R. Chaudhry

**Affiliations:** 1 Department of Neurosurgery, Friedrich-Alexander University (FAU) of Erlangen-Nürnberg, D-91054 Erlangen, Germany; yavor.bozhkov@uk-erlangen.de (Y.B.); natalie.kremenevski@uk-erlangen.de (N.K.); sebastian.brandner@uk-erlangen.de (S.B.); Michael.Buchfelder@uk-erlangen.de (M.B.); 2 Division of Functional Neurosurgery and Stereotaxy, Friedrich-Alexander University (FAU) of Erlangen-Nürnberg, D-91054 Erlangen, Germany; 3 Institute of Medical Radiology, Clinic St. Pölten, Karl Landsteiner University of Health Sciences, 3500 St. Pölten, Austria; Andreas.Stadlbauer@stpoelten.lknoe.at; 4 Department of Basic Medical Sciences, Shifa College of Pharmaceutical Sciences, Shifa Tameer-e-Millat University, Islamabad 44000, Pakistan; shafqatrasul@yahoo.com

**Keywords:** leptin, immune-metabolism, brain tumors therapy, glioblastoma, systemic inflammation, metformin, brain tumor diagnosis

## Abstract

**Simple Summary:**

Despite recent advances in molecular brain tumor therapies, glioblastoma multiforme remains a diagnostic and therapeutic challenge with, in most cases, unfavorable outcome. Leptin and related mediators of immune-metabolic traffic have attracted increased recognition in the past decade in brain tumor biology, in particular potential implications in the diagnosis and treatment of recurrent and newly diagnosed high and low grade gliomas. Randomized controlled trails are on the way to elaborate the role of leptin and its receptor ObR by targeting and using antidiabetic drugs known to interact with distinct pathways associated with leptin signaling. To date, most of the findings in clinical studies remain preliminary and of heterogenous character, although experimental studies have underpinned the relevance of leptin and ObR in the pathophysiology of brain tumors in general.

**Abstract:**

Leptin has been recognized as a potential tumor growth promoter in various cancers including cranial tumor pathologies such as pituitary adenomas, meningiomas and gliomas. Despite recent advances in adjunctive therapy and the established surgical resection, chemo- and radiotherapy regimen, glioblastoma multiforme remains a particular diagnostic and therapeutic challenge among the intracranial tumor pathologies, with a poor long-term prognosis. Systemic inflammation and immune-metabolic signaling through diverse pathways are thought to impact the genesis and recurrence of brain tumors, and glioblastoma multiforme in particular. Among the various circulating mediators, leptin has gained especial diagnostic and therapeutic interest, although the precise relationship between leptin and glioblastoma biology remains largely unknown. In this narrative review (MEDLINE/OVID, SCOPUS, PubMed and manual searches of the bibliographies of known primary and review articles), we discuss the current literature using the following search terms: leptin, glioblastoma multiforme, carcinogenesis, immunometabolism, biomarkers, metformin, antidiabetic medication and metabolic disorders. An increasing body of experimental evidence implicates a relationship between the development and maintenance of gliomas (and brain tumors in general) with a dysregulated central and peripheral immune-metabolic network mediated by circulating adipokines, chemokines and cellular components, and in particular the leptin adipokine. In this review, we summarize the current evidence of the role of leptin in glioblastoma pathophysiology. In addition, we describe the status of alternative diagnostic tools and adjunctive therapeutics targeting leptin, leptin-receptors, antidiabetic drugs and associated pathways. Further experimental and clinical trials are needed to elucidate the mechanism of action and the value of immune-metabolism molecular phenotyping (central and peripheral) in order to develop novel adjunctive diagnostics and therapeutics for newly diagnosed and recurrent glioblastoma patients.

## 1. Introduction

In addition to its canonical role in immune-metabolic signaling (satiety and weight loss/gain), leptin is well-known to promote cellular proliferation, survival and migration through its associated receptor (ObR) and related pathways under physiological conditions, as well as during the initiation and progression of cancers of different organ systems (breast, lung, prostate, brain). Leptin/ObR activities have been characterized as anti-apoptotic, proliferative and potentially pro-angiogenetic [1,2]. In particular, data from experimental (in vivo and in vitro) and human studies indicate that the leptin/ObR axis and its degree of expression may differ depending on brain tumor grade [3,4,5,6]. For instance, compared to low-grade gliomas and other brain tumor entities, glioblastoma multiforme (GBM) cells display a relatively high leptin and ObR expression profile, suggesting a role for the leptin/ObR ratio in the development and recurrence of high-grade, aggressive gliomas (glioblastoma; cell growth, invasion, migration) [1].

Leptin is a polypeptide hormone (~16 kDa consisting of 167 amino acids with an N-terminal secretory-signal sequence of 21 amino acids) that is primarily secreted from adipose tissue and plays an essential role in energy balance by regulating the homeostatic mass of adipose tissue and controlling food intake via hypothalamic nuclei (arcuatus nucleus of the hypothalamus). Leptin and ObR are widely distributed and expressed in the brain and in a broad range of tissues including skeletal muscle, ovaries, placenta and stomach [7,8,9]. Leptin is encoded by the LEP gene on chromosome 7q31.3 in humans (murine analogue: ob gene), and its principal site of action is in the brain, where it mainly acts on the hypothalamus to promote the activity of anorexigenic POMC (Pro-opiomelanocortin) neurons and inhibit orexigenic NPY/AgRP (Neuropeptide Y/Agouti-related protein) neurons in the arcuate nucleus [7,8,9]. In order to regulate metabolic hemostasis, leptin and its receptor ObR directly interact with the arcuate nucleus (ARC) of the hypothalamus, to be more specific, the AgRP-neurons (activation of food intake) and the POMC neurons (inhibition of food intake). Depending on the specific subunit of receptors, AgRP dysfunction leads to emaciation, starvation and death, while alteration of the POMC pathway leads to obesity. In addition, experimental data indicates that leptin is relevant for locomotor activity via melanocortin-expressing neurons of the hypothalamus projecting to striatal dopaminergic neurons [10,11,12]. AgRP projects to the paraventricular nucleus of the hypothalamus and the dorsomedial hypothalamus, which in turn interplay with extra-hypothalamic circuits such as the locus ceruleus, the periaqueductal grey, the parabrachial nucleus and the sympathetic premotor neurons of the brainstem relevant for the central regulation of adipose tissue thermogenesis. Although the precise mechanism remains to be clarified, experimental studies indicate that leptin enters the brain via different entry gates, first, via endothelial cells of the blood-brain-barrier (BBB), second via endothelial cells of the blood-cerebrospinal fluid (CSF) barrier of the choroid plexus, and lastly via stretched cells (ependymoglial cells) coined tanycyte (tight junctions) of the medio-basal hypothalamus, forming a barrier between CSF and the median eminence (ventral proportion of the pars tuberal of the hypothalamus). The receptor for leptin (ObR), expressed in endothelial/epithelial cells and tanycyte of the brain, may act as a pivotal structure for leptin transportation across the BBB and the blood-CSF barrier depending on the isoform [13,14,15,16]. Of note, the central as well as peripheral circadian clock impact the effect promoted by leptin and ObR leading to altered leptin synthesis in adipose tissue and to central leptin resistance [17]. As a pleiotropic hormone, leptin affects other brain regions such as the brainstem, the forebrain and the spinal cord and regulates behavior, thermogenesis, metabolism, the neuro-endocrine axis, immune functions and various other physiological activities [7,8]. Besides its principal activities in the brain, it also directly acts on immune cells in the periphery [7].

ObR belongs to the class I cytokine receptor superfamily and has various isoforms, including a long isoform comprised of extensive cytoplasmic domains that is expressed in nearly all tissues and is responsible for most leptin signal transduction [18]. Signal transduction through this receptor involves tyrosine kinases of the Janus kinase family (JAKs) and signal transducers and activators of transcription (STATs) [9,18]. Following leptin binding to its extracellular domain, ObR dimerizes, leading to a conformational change that activates JAK2, which is non-covalently associated with ObR’s cytoplasmic domain, leading to downstream activation of STAT3 [7,19]. Activated STAT3 then upregulates the transcription of target genes, including SOCS3 (suppressor of cytokine signaling-3), which acts in a negative feedback loop to inhibit leptin signaling [7,19].

ObR activation also leads to the phosphorylation and activation of STAT5 and other signaling pathways such as ERK (Extracellular signal regulated kinase), MAPK (Mitogen activated protein kinase), mTOR (mammalian Target of Rapamycin) and Src homology domain 2 (SH-2)-domain containing protein 1B (SH2B1)/insulin receptor substrate (IRS)/phosphoinositide-3-kinase (PI3K) pathway [19,20].

Given the increasing evidence, we sought to highlight and discuss the relevant literature covering the relationships between and potential diagnostic value of leptin, ObR and the complex biological characteristics of GBM. In addition, this narrative review will discuss current and future therapeutic considerations targeting leptin’s immune-metabolic activity (antidiabetic drugs).

## 2. Material and Methods

PubMed, MEDLINE/OVID, and SCOPUS along with manual searches of the bibliographies of original and review articles from 1990–2020 were used to identify relevant literature using the following terms: leptin/ObR, glioblastoma (newly-diagnosed and recurrent), carcinogenesis, immune-metabolism, biomarkers and metabolic disorders. The primary criteria for inclusion in our review were the following terms: glioblastoma multiforme—brain tumor-leptin—leptin receptor–immune-metabolism—molecular phenotyping experimental and in-human studies–metformin–antidiabetic drugs.

## 3. Relevant Pathways for Leptin-Induced Glioma Genesis and Recurrence

**Figure 1:**
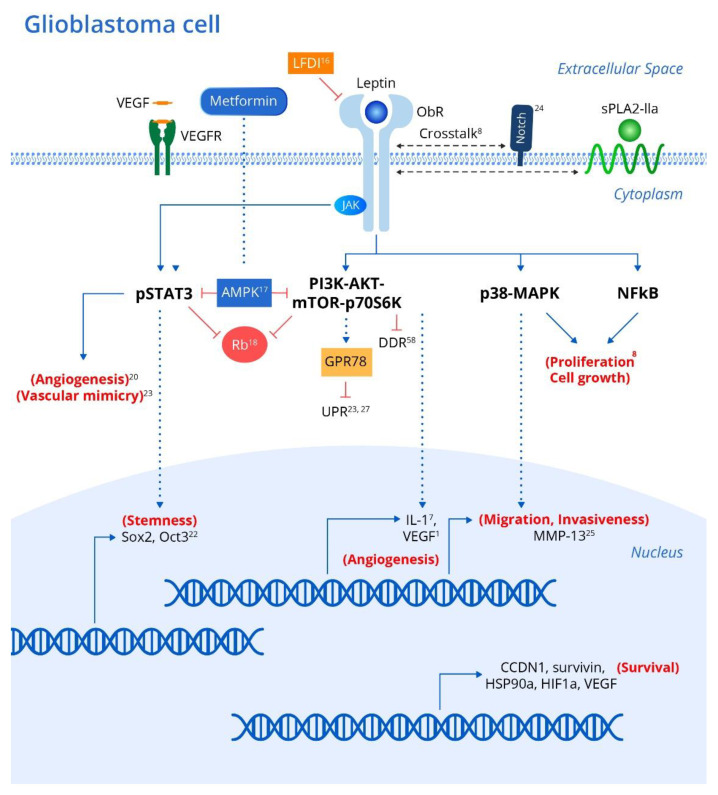
Leptin and associated signaling pathways that influence the development and recurrence of gliomas. Integrating metabolic and inflammatory pathways, the Leptin/ObR axis initiates a coordinated array of pro-tumor effects. Binding of leptin to the ObR receptor activates a variety of downstream signaling events, culminating in the expression of genes that promote cellular proliferation, growth, survival, invasion, stem-cell-like behavior, and increased angiogenic activity in brain tumor cells and associated tissue. Leptin’s angiogenic effects via the JAK/STAT and PI3K/AKT/mTOR pathways are particularly noteworthy given the extensive micro-vascularization of gliomas that arises from tumor cell vascular mimicry. ^xx^ corresponding references (superscript). Abbreviations: ObR (leptin receptor); JAKs (Janus kinase family); STATs (signal transducers and activators of transcription); SOCS3 (suppressor of cytokine signaling-3); MAPK (Mitogen activated protein kinase); mTOR (mammalian Target of Rapamycin); PI3K (IRS)-phosphoinositide-3-kinase); sPLA2-IIa (phospholipase A_2_-IIa); AMPK (adenosine monophosphate associated protein kinase); Rb (retinoblastoma protein); CCDN1 (cyclin D1); survivin (BIRC5); HSP90A (heat shock protein); HIF1A (hypoxia inducible factor); VEGF (vascular endothelial growth factor); MMP-13 (matrix metalloproteinase-13); UPR (unfolded protein response); GRP78 (chaperone molecule; 78 kDa glucose regulated protein); DDR (DNS damage response).

Genetic polymorphisms in either leptin or its receptor are linked to increased risk and progression of various cancers including oral, prostate and breast cancers [7]. In addition, leptin and its receptor have been shown to be expressed in various brain tumor pathologies [20,21]. Leptin has been shown to upregulate various intracellular signaling pathways linked to excessive proliferation, growth, migration, and invasion in various glioblastoma cell lines (Figure 1) [8] including NF-κB, p38-MAP Kinase, JAK/STAT3 and PI3K/Akt/mTOR/P70S6K. Interestingly, crosstalk between leptin and soluble Phospholipase A_2_-IIa enhances proliferation and migration in astrocytoma cell lines via the PI3K/Akt/mTOR/P70S6K pathway (Figure 1) [8]. Furthermore, ObR expression in glioblastoma cells imparts progenitor/stem cell-like properties through STAT3-mediated upregulation of the pluripotency-associated SOX2/OCT4 signaling axis, which underlies the self-renewal properties of glioma cells and resistance to drugs such as temozolomide (Figure 1) [18]. Interestingly, upregulation of adenosine monophosphate-associated protein kinase (AMPK) following metformin administration abrogates the leptin-induced growth and migration of glioblastoma cells, also mediated by downregulation of STAT3 and Akt/PKB serine threonine kinase (Figure 1) [22].

As mentioned above, STAT3 and Akt/PKB are known to be consistently upregulated in various glioblastoma cell lines due to leptin/ObR overexpression, which drives dysregulation of the cell cycle suppressor Rb (retinoblastoma protein), thus promoting uncontrolled cellular proliferation in the absence of normal suppressive mechanisms (Figure 1) [23].

Antagonism of ObR in glioblastoma cell lines peptide inhibits proliferation and invasiveness, and is associated with downregulation of leptin-responsive genes such as cyclin D1 (CCDN1), survivin (BIRC5), heat shock protein (HSP90A), hypoxia inducible factor (HIF1A) and vascular endothelial growth factor (VEGF) [19]. Intriguingly, the stem cell behavior in glioblastoma cell lines has been associated with upregulation of NOTCH and its downstream signaling pathways, as evident by increased expression of the NOTCH isoforms (NOTCH1, NOTCH2, NOTCH3, NOTCH4) and NOTCH target genes (HES1, survivin) (Figure 1) [24]. Selective LFDI-mediated antagonism of ObR abrogates NOTCH signaling, suggesting a critical role of leptin/ObR in the modulation of NOTCH-dependent growth, proliferation and migration of glioblastoma cells [24], and a previous study suggested that this invasiveness and migration were driven by increased levels of matrix metalloproteinase (MMP)-13 via the MAPK pathway (Figure 1) [25].

One hallmark of all cancers is the upregulation of mechanisms leading to sustained angiogenesis. Interestingly, transformation of low-grade gliomas into the more aggressive and lethal glioblastoma multiform (GBM) is associated with overexpression of intra-tumoral leptin as well as increased angiogenesis [23]. Leptin released from glioma cells partially contributes to angiogenesis, as evaluated by induced tube formation capacity in human umbilical vein endothelial cells (HUVECs) via STAT3 phosphorylation [23].

Interestingly, VEGF also activated angiogenesis through STAT3 phosphorylation, and co-inhibition of the receptors for both leptin and VEGF suppressed angiogenesis in HUVECs induced by conditioned medium from glioma cells more than either antagonist alone (Figure 1) [23]. Leptin synergizes with and even increases the levels of VEGF in various cancers, probably through MAPK- and PI3K-mediated upregulation of IL-1 [1]. In addition to typical tumor angiogenesis, the extensive micro-vascularization of glioblastomas also relies on vasculogenic mimicry (Figure 1) [26], in which tumor cells rather than endothelial cells promote new vessels to augment tumor blood supply. Intriguingly, glioblastomas with high ObR expression displayed more extensive vasculogenic mimicry, which was associated with poor prognosis [26]. Cancer cells also acquire growth-sustaining and antiapoptotic mechanisms. Interestingly, transfection of neuroblastoma cells with ObR protected against the endoplasmic reticulum (ER) stress response via the unfolded protein response (UPR) by upregulating the chaperone molecule GRP78 (78 kDa glucose regulated protein) [27]. While sustained ER stress induces apoptosis, GRP78 induction by leptin was not associated with prolonged UPR signaling (which typically induces apoptosis), and is instead dependent on the PI3K-Akt-mTOR pathway, thus protecting the cells against apoptosis [23]. Chaperones are known to promote tumor growth and survival, and GRP78 upregulation downstream of leptin may also underlie glioblastoma cell survival, which requires further investigation (Figure 1) [27].

## 4. Associations between Brain Tumor Entities and Treatment Outcome with the Metabolic State

Increasing evidence supports a link between obesity and associated metabolic disorders with carcinogenesis in a variety of organ systems. In contrast to previous paradigms, white adipose tissue (WAT) is now considered not only to be an energy storage system, but also to act as an active endocrine organ capable of communicating with central and peripheral inflammatory mediators that promote the development of a wide variety of neurological, psychiatric, metabolic and oncological diseases [28].

WAT cells synthetize leptin and related immune-metabolic mediators, which in turn drive cancer development, maintenance and recurrence. The main biological characteristics of leptin have been described as promoting inflammation, proliferation, anti-apoptosis, cell migration, differentiation and invasion [28]. In a meta-analysis, Sergentanis et al. assessed the potential association between overweight/obesity and the likelihood of developing brain tumors, gliomas and meningiomas [29]. Notably, a strong correlation was detected between overweight/obese females and the risk of brain/CNS tumors, meningiomas and gliomas, and a trend towards increased aggressiveness was observed. In contrast, obese male subjects were at increased risk for meningiomas only, indicating gender-associated differences in immune-metabolic pathways [29]. These findings have led researchers to hypothesize whether hypertension, diabetes mellitus and cardiovascular disease–together under the umbrella term “metabolic disorder”–may help predict diagnosis and guide treatment decisions for brain tumors in adults, as well as in children [30,31]. Based on the increasing evidence for an overall increased risk of cancer with obesity, Chambless et al. retrospectively determined the prognostic value of pre-existing obesity and type 2 diabetes mellitus (T2DM) in 171 patients with a confirmed diagnosis of high-grade glioma (HGG) and found significant correlations of increased age, diabetes mellitus and obesity with decreased survival and poor outcome. These findings support the potential utility of a diagnostic and therapeutic framework using molecular inflammatory phenotyping of immune-metabolism signaling, and leptin biology in particular [30]. It is noteworthy that, in addition to obesity, a dysregulated metabolic state associated with underweight may predict poor outcome for HGG patients. Siegel et al. evaluated over- and underweight HGG subjects for overall survival and observed that both metabolic conditions predicted poor outcome, in contrast to other studies that found no differences in therapeutic responsiveness [32]. Various experimental evidence has shown that leptin, ObR and leptin-related genes have been identified in human tissue from various brain tumors including gliomas, metastasis, oligodendrogliomas, hormone-secreting and non-functioning pituitary adenomas.

Hence, further clinical research is needed in order to elucidate the specific characteristics and biological role of the leptin/ObR axis relative to various brain tumor entities [33,34].

Knerr et al. investigated the gene expression profile of the leptin/ObR system in relation to proliferative activity in astrocytoma and glioblastoma human samples using reverse transcriptase-polymerase chain reaction (RT-PCR). Leptin/ObR components were detected in all brain tumor subgroups, suggestive of a broad functional role of leptin in brain tumor biology [33].

## 5. Molecular Evidence Supporting Leptin as a Potential Diagnostic Factor in Glioma Patients

Leptin/ObR are present in a broad range of brain tumors, with the level of expression associated with the degree of malignancy, raising the possibility that phenotyping of peripheral circulating leptin/ObR may be a useful strategy for predicting tumor grade. Using immunohistochemistry and real-time PCR, leptin/ObR expression was found in 55% and 61%, of 87 human brain tumor biopsies. Furthermore, in the biopsy samples in which leptin was detected ObR co-localization was confirmed in ~80%. These findings were in part specific to glioblastoma and anaplastic astrocytomas, while lower expression rates were found in low-grade astrocytoma (LGG) and gangliogliomas (weak expression of tumor cells: 25% versus moderate expression of tumor cells: 30% versus strong expression of tumor cells: 30% [35,36]). Limitations on these observations of the various tumor grades are the small number of tissue samples assessed and the lack of follow-up evaluation. It is noteworthy that leptin/ObR were co-expressed with associated signaling pathway markers such as STAT3 and Akt, which in turn are involved in tumor progression and invasion. ObR-positive glioblastoma cells were responsive to leptin, which promoted cell growth and activated the STAT3/Akt cascade [35].

The associations between leptin/ObR and glioblastoma have paved the way towards diagnostic approaches (primary or recurrent glioblastoma) using whole-serum infrared spectroscopy combined with immunoassays (cytokines, angiogenesis markers) in serum samples.

In general, several advantages are associated with such diagnostic tools, including rapid testing, the ability to monitor glioblastoma therapy responses, and the possibility of early detection of tumor recurrence and progression [37,38,39]. However, the specificity and the sensitivity of such diagnostic methodologies remain to be established, an issue of ongoing experimental and clinical research.

Hands et al. developed and applied a combined diagnostic approach (spectroscopy plus immunoassays) to assess angiopoietin, follistatin, platelet-derived growth factor-BB (PDGF-BB), platelet endothelial cell adhesion molecule-1 (PECAM-1), interleukins (IL-2, IL-4, IL-6, IL-8, IL-10), tumor necrosis factor (TNF-α), VEGF, hepatocyte growth factor (HGF) and leptin, in serum samples of 50 glioblastoma patients compared to healthy controls [37]. In view of the dynamic and highly complex nature of the relation between leptin/ObR and glioma biology, combined multi-stage diagnostics have been recommended in order to appropriately distinguish, interpret and most importantly enhance the sensitivity/specificity ratio of serum sample analytics [37,38,39,40].

Significant differences between glioblastoma samples and healthy controls were observed for angiopoietin, follistatin, HGF, IL-8, PDGF-BB, PECAM-1 and leptin, with specificity ranging from 59–81% and sensitivity from 40–88%, while infrared-spectroscopy reached 100% sensitivity and 87.5% specificity. In particular, leptin immunoassays displayed a lower sensitivity of 40%, and specificity of 74%. This interesting diagnostic protocol would potentially offer several advantages, such as more rapid diagnosis confirmation (less than 5 h) and avoidance of surgical interventions and hospitalization. In cases of incongruent findings between the spectroscopy and immunoassays, established protocols (surgery, imaging, immunohistology) would be used to provide the diagnosis. Of note, the potential impact of obesity–BMI was not explicitly included in this approach [37].

## 6. Current Therapeutic Concepts for Glioblastoma Multiforme Targeting the Leptin/ObR Axis

To date, the standard of care for newly diagnosed glioblastoma comprises surgical resection, temozolomide (TMZ) and radiation, which give an average overall survival of 15 months. Recurrent glioblastoma is mainly treated with resection (if applicable) and chemotherapeutics (TMZ, nitrosoureas, bevacizumab), achieving a 12-month overall survival in approximately 14% of patients [41]. Currently, there are various drugs available that target specific glioblastoma pathways (growth, invasion, migration) including but not limited to tyrosine kinase inhibitors (EGFR, mTOR, VEGF), NF-κB modulators, immunotherapeutics and nitrosoureas.

Monoclonal antibodies (MABS), immune checkpoint inhibitors, vaccines and adaptive cell therapy/chimeric antigen receptor (CAR) T cells target immunological pathways relevant for glioblastoma. Although some encouraging findings have been reported, there remains substantial development for additional pharmacotherapeutics targeting novel molecular signals, among which the leptin/ObR axis deserves special attention as an addendum to established protocols [41].

Notably, caution is advised when interpreting the findings highlighted in this review, as a disrupted leptin/ObR axis may be either a consequence (e.g., secondary leptin-resistance due to hypothalamic lesion or obesity) or a promoter (local, systemic and exogenous leptin promotes tumor-genesis) of brain tumors, while both situations may show overlapping temporal dynamics [40].

Metformin, a first-line oral antidiabetic drug, has received increasing attention for its anti-tumor properties observed in clinical in-human trials. The therapeutic value of metformin for cancer biology and treatment outcomes in type 2 diabetes has been an issue of ongoing discussion. In their meta-analysis of observational cohort human studies, Farmer et al. found considerable biases in the interpretation of published results, such as inappropriate statistical methods neglecting the influence of time-varying treatment and previously applied treatment protocols. Furthermore, those few studies identified as having a robust design did not find any associations between metformin and cancer outcome, suggesting that metformin may not to be protective against cancer overall [42]. Contrasting observations have been made in otherwise difficult-to-treat lung cancers, such as non-small cell lung cancer (NSCLC) and squamous cell lung carcinoma, which suggest a potential protective role of metformin in cancer, notable given the limited available therapeutic options in such circumstances [42]. Most of these findings are derived from in vivo and in vitro preclinical studies and observational in-human trials and must be validated in randomized controlled trials (Table 1) [42,43,44]. It is worth noting that most dosages of metformin/biguanides used in human cancer trials represent “conventional” concentrations applied in diabetes treatment. Consequently, higher metformin doses should be evaluated in further randomized-controlled trials, considering that the drug’s known toxicology may have a maximal impact on cancer biology and therefore be of relevance for cancer treatment [45]. In particular, this needs to be addressed in glioblastoma patients, as most receive glucocorticoids, which are well known to elevate blood glucose levels and therefore may increase the risk of poor outcome (survival rate, progression free survival) [43]. The interplay of glioma cells with a hyperglycemic state may interfere with established glioblastoma therapeutic strategies such as surgical resection, chemo-therapy and radiotherapy [46,47,48]. This hypothesis mainly derives from experimental in vivo and in vitro studies that have suggested a potential indirect and direct effect of metformin on proliferation, migration, treatment resistance and cell growth inhibition via a broad range of signaling pathways [49,50,51,52,53,54,55,56,57,58,59,60].

Given the associations of obesity/diabetes and glucose-lowering antidiabetic agents (e.g., metformin) with glioblastoma outcomes, metformin (drug re-purposing) is currently under investigation as an adjunctive therapy in an established pharmacotherapeutic/radiotherapy and behavioral (diet) treatment in LGG and GBM patients in several ongoing RCTs [(NCT01430351–phase 1–n = 144 patients), (NCT02496741–phase 1b–n = 15 patients), (NCT02149459–phase 1–n = 18 patients), (NCT02780024–phase 2–n = 50 patients), (NCT03243851–phase 2–n = 108 patients) and (NCT03151772–phase 1–n = 40 patients)] (Table 1). This drug repurposing of metformin aims to inhibit the metabolic energy supply relevant for brain tumor development and/or recurrence. However, these clinical studies are challenging because GBM patients receive a broad range of drugs that may impact energy supply/glucose metabolism (metformin, insulin, corticosteroids, sulfonylureas), and these may be altered over the treatment course, limiting the ability to identify potential associations related to a single drug (e.g., metformin) [61,62,63,64,65,66].

Among its other physicochemical characteristics, metformin can penetrate the blood-brain-barrier (BBB) to distribute across the CNS, making metformin particularly attractive for CNS malignancies. The precise mechanism by which metformin exerts its effects remains largely unknown, but a direct suppressive effect on cancer cells and an indirect effect via decreased glucose, insulin and insulin-like growth factor have been suggested. With regard to glioblastoma-associated signaling, metformin may interfere with AMPK activity, which in turn modulates (suppression) mTOR signaling relevant for tumor growth and invasion, as regulated during tumor development via the DNS damage response (REDD1) pathways and oxidative stress (Figure 1) [61].

Given that isocitrate dehydrogenase (IDH) 1/2-mutated gliomas (II/IV) have been associated with poor outcomes, and that IDH 1/2-glioma cells promote the synthesis of so-called oncometabolites such as D2-hydroxyglutamate (D-2HG), which are potentially responsive to metformin, an ongoing dose-finding phase 1b/2 trial determined the efficacy and toxicity of a combined drug protocol consisting of metformin and chloroquine (CQ) in IDH1/2-mutated HGG, high-grade chondrosarcoma and intrahepatic cholangiocarcinoma. In order to establish the recommended dosage, metformin hydrochloride (MFRMN)/CQ was applied using a dose-escalation protocol (NCT02496741) [57]. The secondary outcome measures included an assessment of serum concentrations, computertomography (CT)/magnet resonance imaging (MR) evaluation and assays of D-2HG levels in order to characterize the pharmacokinetic characteristics, treatment response and toxicity of both antitumor agents (MFRMN–CQ). Aside from the study protocol, preliminary findings have not yet been published (Table 1) [62].

Seliger et al. retrospectively analyzed the potential relationship between MFRMN use and tumor response (survival) in a pooled cohort from the Avastatin in Glioblastoma trial (AVAglio; NCT 00943826; 921 participants), the CENTRIC study (NCT 00689221; 545 participants) and the CORE study (NCT 00813943; 265 participants). In contrast to previously reported beneficial effects of antidiabetics, MFRMN application was not found to be associated with an improved OF or PFS during adjunctive standard of care GBM treatment (TMZ/RT), indicating that additional antidiabetic drugs may not have a considerable impact on overall survival rate (OS)/progression free survival rate (PFS). Notably, a non-significant association between baseline MFRMN use and GBM survival was observed at baseline, but not in the TMZ/RT phase.

Furthermore, GBM responsiveness did not differ for pre-existing diabetic and/or hyperglycemic participants compared to those without metabolic disturbances [63].

A subsequent retrospective cohort analysis conducted by Seliger et al. observed the opposite effect, as MFRMN was significantly associated with increased overall survival (OS) and progression-free survival (PFS) in grade III gliomas (n = 231 subjects), while no improvement was observed for the HGG subgroup (n = 862 subjects), supporting the hypothesis that IDH 1/2-MT status and a lower grade may increase the likelihood of response to metabolically active drugs (Table 1) [64]. These findings were in part confirmed by an additional retrospective GBM cohort study. While the univariate analysis demonstrated an increased PFS in diabetic GBM patients with adjunctive MFRMN medication, there were no associations in the multivariate assessment [65].

A large retrospective study detected a trend towards improved OS in diabetic GBM patients under MFRMN therapy (not significant), while hyperglycemia was associated with a worse OS. Most interestingly, differences in OS occurred in the subgroup with reduced corticosteroid (dexamethasone) intake; steroid use was strongly correlated with a poorer OS rate [66]. These findings were in concordance with previously published in vivo and in vitro experimental data, highlighting the critical need to re-examine adjunctive antidiabetic drug regimens for glioblastoma treatment. As noted previously, modulation of the neuro-immune axis has become an increasingly important therapeutic approach in the development of novel, alternative drug protocols for newly diagnosed and recurrent glioblastoma [62].

In a patient-derived GBM preclinical model, IL-17A inhibitors increased overall survival and decreased tumor growth/tumor hypoxia, angiogenesis, VEGF expression, cell proliferation, leptin expression and adipogenesis compared to control samples. However, clinical studies have not been performed using IL-17A inhibitors [67].

Lastly, environmental enrichment (EE–physical/social activity), in particular eustress (beneficial) and its opponent distress (inactivity–social isolation), has been shown to delay (eustress) or to promote (distress) disease progression in a variety of disorders (Parkinson, Alzheimer) and malignancies (cancers of the breast, prostate, colon and melanoma) [1]. Briefly, EE along with psychological (behavioral) therapies led to increased levels of brain-derived neurotrophic factor (BDNF), which in turn decreases systemic leptin levels through sympathetic transmission (via β-adrenergic receptors on WAT cells). At this stage, the impact of EE on glioblastoma via the hypothalamic-sympathetic-WAT route remains to be elucidated in human cells [1].

## 7. Conclusions and Future Targeted Research

In conclusion, leptin and its receptor ObR appear to play a major role in glioblastoma pathophysiology by inhibiting apoptosis and promoting cell growth, invasion, angiogenesis and proliferation. Increasing evidence from experimental studies (e.g., glioblastoma C6 cells lines) and some clinical studies have led to randomized-controlled trials incorporating inhibitors of the leptin/ObR axis as adjunctive therapy with established protocols in newly diagnosed and recurrent glioblastoma. To what extent modulation of the immune-metabolic axis may become a complementary diagnostic/therapeutic tool remains to be elaborated. At this stage, caution is warranted in the interpretation of these data, as the diagnostic and therapeutic value of leptin and ObR remains uncertain due to the conflicting and biased nature of the available data. In parallel, further experimental studies are needed to elucidate the precise mechanism of action of the leptin/ObR system in the context of different brain tumor pathologies and grades. The strategy of “bench to bedside and back” may be the most appropriate approach to develop novel glioblastoma therapy protocols to iteratively improve diagnosis and enhance glioma survival.

## Figures and Tables

**Table 1:** Summary of In-Human GBM studies targeting leptin and associated mediators of immunometabolic signaling.

Year	Study Design	Patients	Treatment Protocol	Primary Endpoint	Secondary Endpoint	Outcome/Status quo	SAE/AE
**2020 [61]**	Phase 1 NCT01430351 RCT	144	TMZ+MFRMN TMZ+MFLOQ TMZ+MEMNT *Newly GBM* after SOC treatment	Safety/Tolerability	PFS (6,12,18 m) OS	Active/Not recruiting	---
**2020 [62]**	Phase 1b/2 NCT02496741 Open Label	15	MFRMN+CQ *Newly GBM IDH1/2MT-pos*	Efficacy/Toxicity (maximum dose)	Pharmacokinetic IDH1/2MT assays Tumor response	---	---
**2020 [61]**	Phase 1 NCT02149459 Open Label	18	MFRMN+RT *Recurrent GBM*	Safety/Tolerability	Tumor response Energy Metabolism (glucose, insulin)	Recruiting	---
**2020 [61]**	Phase 2 NCT02780024 Open Label M-HARTT STUDY)	50	1.TMZ+MFRMN 2.TMZ+RT 3.TMZ alone *Recurrent GBM*	OS	Toxicity/Tolerability	Recruiting	---
**2020 [61]**	Phase 2 NCT03243851 RCT	108	TMZ+MFRMN Versus TMZ+Placebo *Recurrent/Refractory GBM*	PFS	Tumor response/Tumor control probability PFS (6m) OS (6m) EORTC QLQ-C30 EORTC QLQ-BN20	Recruiting	---
**2020 [61]**	Phase 1 NCT03151772 Open Label	40	Disulfiram MFRMN *Newly/Recurrent GBM*	Bioavailability at time of surgery	---	Recruiting	---
**2020 [63]**	Retrospective Pooled Analysis (AVAglio, CENTRIC, CORE trials)	1731	MFRMN+TMZ/RT (with/without diabetes) *Newly GBM*	OS PFS	---	No significant association between diabetes, metformin use/glucose levels with OS or PFS	---
**2019 [64]**	Retrospective Pooled Analysis	1093	Prognostic value of MFRMN *(Grade III glioma/GBM)*	OS PFS	---	1. Significant improved OS/PFS for III glioma 2. No OS/PFS improvement for IV glioma	---
**2015 [65]**	Retrospective Analysis	276	Prognostic value of diabetes/glucose level/MFRMN *(Newly GMB)*	OS PFS	---	1. Hyperglycemia and corticosteroid correlated with decreased OS/PFS 2. No correlation of OS/PFS with diabetes 3. MFRMN use associated with improved PFS	---
**2013 [66]**	Retrospective Analysis	988	Prognostic value of Corticosteroid/MFRMN *(Newly GMB)*	OS		1.Improved OS in MFRMN use 2. Decreased OS in hyperglycemia (corticosteroid)	---

**Abbrev.:** metformin hydrochloride (MFRMN)–mefloquine (MFLOQ)–memantine (MEMTN)–chloroquine (CQ)–glioblastoma (GBM)–high-grade glioma (HGG)–low-grade glioma (LGG)–overall survival (OS)–progression-free survival (PFS)–radiotherapy (RT)–months (m)–European Organization for Research and Treatment of Cancer Quality of Life Questionnaire (EORTC-QLQ)–Brain Tumor Module (EORTC-QLQ-BN20)–isocitrate dehydrogenase (IDH).

## Abbreviations

ObR	leptin receptor
GBM	glioblastoma multiforme
HGG	high-grade glioma
LGG	low-grade glioma
T2DM	type 2 diabetes mellitus
TMZ	temozolomide (TMZ)
POMC	pro-opiomelanocortin
NPY/AgRP	neuropeptide Y/agouti-related protein
ARC	arcuate nucleus
BBB	blood-brain-barrier
CSF	cerebrospinal fluid
JAKs	Janus kinase family
STATs	signal transducers and activators of transcription
SOCS3	suppressor of cytokine signaling-3
ERK	extracellular signal regulated kinase
MAPK	mitogen activated protein kinase
mTOR	mammalian target of Rapamycin
SH-2	Src- homology domain 2
SH2B1	domain containing protein 1B
(IRS)	insulin receptor substrate
(PI3K)	phosphoinositide-3-kinase
WAT	white adipose tissue
RT-PCR	reverse transcriptase-polymerase chain reaction
PDGF-BB	platelet-derived growth factor-BB
PECAM-1	platelet endothelial cell adhesion molecule-1
IL	interleukins
TNF-α	tumor necrosis factor
VEGF	vascular endothelial growth factor
HGF	hepatocyte growth factor
MABS	monoclonal antibodies
CAR	chimeric antigen receptor
D-2HG-D2	hydroxy-glutamate
CQ	chloroquine
IDH	isocitrate dehydrogenase
BDNF	brain-derived neurotrophic factor
MFRMN	metformin hydrochloride
MFLOQ	mefloquine
MEMTN	memantine
PI3K-(IRS)	phosphoinositide-3-kinase
sPLA2-IIa	phospholipase A_2_-IIa)
AMPK	adenosine monophosphate associated protein kinase
Rb	retinoblastoma protein
CCDN1	cyclin D1
BIRC5	survivin
HSP90A	heat shock protein
HIF1A	hypoxia inducible factor
MMP-13	matrix metalloproteinase-13
UPR	unfolded protein response
GRP78	chaperone molecule; 78 kDa glucose regulated protein
DDR	DNS damage response
NSCLC	non-small cell lung cancer
RCT	randomized controlled trial
EE	environmental enrichment
OS	overall survival
PFS	progression-free survival
RT	radiotherapy
EORTC-QLQ	European Organization for Research and Treatment of Cancer Quality of Life Questionnaire Brain Tumor Module (EORTC-QLQ-BN20).
